# Assessing Household’s Municipal Waste Segregation Intentions in Metropolitan Cities of Pakistan: A Structural Equation Modeling Approach

**DOI:** 10.1007/s10661-023-11685-w

**Published:** 2023-09-14

**Authors:** Tanzila Akmal, Faisal Jamil, Muhammad Haseeb Raza, Cosimo Magazzino, Babar Hussain

**Affiliations:** 1 National Business School, The University of Faisalabad, Faisalabad, Pakistan; 2 School of Social Sciences and Humanities, National University of Sciences and Technology (NUST) Islamabad, Islamabad, Pakistan; 3 Department of Agribusiness & Applied Economics, MNS University of Agriculture, Multan, Pakistan; 4 Department of Political Science, Roma Tre University, Rome, Italy; 5 International Islamic University, Islamabad, Pakistan

**Keywords:** Household waste management, Segregation intentions, Socio-psychological aspects, Institutional factors, Situational factors, Structural Equation Modeling, Pakistan

## Abstract

There is a growing concern about inappropriate waste disposal and its negative impact on human health and the environment. The objective of this study is to understand household waste segregation intention considering psychological, institutional, and situational factors simultaneously. Insights into the motivations of household waste segregation drivers may assist in a better knowledge of how to pursue the most efficient and effective initiatives. For this purpose, data from a representative sample comprising 849 households is obtained from the twin cities of Islamabad and Rawalpindi (Pakistan). The empirical analysis employs a Structural Equation Modeling (SEM) approach, showing that policy instruments have significant direct and indirect impacts on households’ segregation intentions. The results highlight that government policy instruments strengthen personal and perceived norms for waste segregation intentions, resulting in an external intervention that would encourage intrinsic motivation. Therefore, policy actions become the main entry point for initiating waste segregation behavior. Public policy must continue to emphasize waste segregation since it may help resource recovery. This is imperative because the environment is a shared resource, and its conservation increases social welfare.

## Introduction

Solid waste has emerged as one of the most critical global environmental issues that strike at the heart of urban amenities (Ashraf et al., 2016; Magazzino & Falcone, 2022). Mainly, household waste is a global issue but has gradually leaped onto the political agenda in developing countries. The core of the issue lies in environmental externalities, and many developed countries have addressed this concern by adopting an Integrated Solid Waste Management System (ISWMS) or advanced recycling (Magazzino et al., 2020). As a result, an ISWMS reduces financial and environmental costs by implementing segregation at the source (Oteng-Ababio, 2014; Soma et al., 2020). Despite the numerous benefits of waste segregation, most developing countries have failed to implement this regulatory measure rigorously. For instance, most cities in Pakistan have been unable to provide an efficient Waste Management System (WMS), resulting in uncontrolled open dumping on vacant areas, ditches, parks, and roadsides. Sustainable household waste management activities include inadequate collection and sorting, legislation to incentivize, and households for a low participation rate in waste segregation and recycling activities.

Due to the increasing population and urbanization, Pakistan faces a solid waste management issue that has recently received immense attention (Bartiaux, 2008). However, approximately 20% of recycling occurs in metropolitan Lahore, which has a relatively organized WMS in the country (Batool et al., 2008). Nevertheless, sustainable household waste management is a growing concern in Pakistan due to inadequate collection, sorting, financial and administrative constraints, flawed legislation, and a low rate of participation in waste segregation and recycling activities (Ashraf et al., 2016).

The impact of regulations on recycling and the minimization of waste decisions has been extensively explored in literature. Recycling behaviors have essentially been linked to three sets of attributes. Situational factors include both enabling and disabling influences such as environmental concerns and environmental knowledge-based (Barr, 2007; Schahn & Holzer, 1990; Zhang et al., 2019). Institutional variables are government-related elements that include recycling policy initiatives such as penalties and rewards to increase people’s recycling rates (Xu et al., 2017). Psychological elements are unique perceptual features that include altruistic motivations to recycle (Barr, 2007; Hopper & Nielsen, 1991). De Young (1986) found that the “intrinsic motivation to act” has a long-term impact on waste management behavior compared to extrinsic motivation behavior. Furthermore, an individual’s belief that environmental challenges threaten well-being may impact their decision to engage in various forms of environmental actions (Segun et al., 1998; Steel, 1996). Chan (1998) argued that self-efficacy can be a crucial determinant of individual recycling behavior. Selman (1996) posited that environmental citizenship is vital in defining individual environmental behavior.

Ofstad et al. (2017) and Xu et al. (2017) proposed that psychology can be applied to understand the process of household responsiveness to waste segregation behavior. Adaptation behavior is fundamentally a cognitive process involving values and beliefs, attitudes and perceptions, personalities, motives, ambitions, and culture; these cognitive elements influence household judgments on the hazardous effects of waste on human health and the environment (Cardwell & Elliott, 2013; Choon et al., 2016; Yadav & Samadder, 2018).

Due to the complexities of this framework, researchers faced difficulty in conceptualizing the problem and seeking its solution. However, it has been shown that the primary link in an established social behavior theory can be used to investigate the relationship between these multiple variables and waste management behavior. Fishbein and Ajzen (1975) employed the Theory of Reasoned Action (TRA) to investigate the link among attitudes, subjective norms, intentions, and behavior. Ajzen (1991) and Boldero (1995) questioned the efficacy of TRA in determining behavioral responses. Fishbein and Ajzen (1975) established a Theory of Planned Behavior (TPB) that extended TRA and implied that an individual’s decision to engage in specific behavior is driven mainly by personal intention. In turn, the intention is affected by three independent constructs: attitudes, subjective norms (social pressure), and perceived behavioral control (ease/difficulty).

TPB has received considerable attention in the literature, asserting that an individual’s choice to participate in a particular behavior is predominantly driven by personal intention (Ajzen, 1991; Bijttebier et al., 2018; Pakpour et al., 2014). Thus, the study’s theoretical model is based on TPB, where intentions guide the households’ actions. Nevertheless, traditional TPB constructs are not always sufficient to facilitate behavioral change; therefore, we investigate the effects of government policy instruments, environmental knowledge, and environmental concerns of households to waste segregation intentions.

In this line, the objectives of the present study are the following:

1. to investigate the direct effect of an extended TPB on households’ waste segregation intentions;
2. to analyze the indirect effect of institutional and situational factors on households’ waste segregation intentions via a TPB.

The participation of households indicating their intentions plays a crucial role in sustainable waste management. Previous research primarily focused on whether various strategies could promote waste recycling and segregation, with little attention paid to the correlation between situational, institutional, and psychological factors as key determinants of individual behavior (Li et al., 2019; Varotto & Spagnolli, 2017). We try to contribute to fill this gap and provide some advances to the existing literature by elucidating the causative linkages among institutional, psychological, and TPB factors in determining household waste segregation intentions in Pakistan. We further investigated how instrumental and psychological factors significantly impact households’ waste segregation intentions. In addition to the traditional TPB, government policy instruments, environmental concerns, and environmental knowledge were considered essential factors in behavioral intention demonstration. The study framework is built on the concept that the intention of household waste segregation cannot be determined without considering the above-mentioned factors. The findings will help in formulating policy options for waste segregation at the source to build a long-term WMS.

The rest of the paper is structured as follows. Section 2 illustrates the extended TPB and explains the theoretical approach. Section 3 describes the sampling methods, item measurement, and reliability measures for the SEM. Section 4 discusses the findings of the analysis. The conclusions are outlined in Section 5. Finally, Section 6 reports policy implications, limitations of the study, and future research.

## Theoretical framework and hypotheses

### The Theory of Planned Behavior

TPB, developed by Ajzen (1985), is primarily used to assess behaviors, social norms, perceived behavioral control, and their impact on the behavioral intentions of individuals. The theory asserts that attitudes (ATT), subjective norms (SN), and perceived behavioral control (PBC) play an essential role in determining household behavioral intentions for waste segregation. Attitudes imply an individual’s positive or negative propensity to enact specific behavior (Ajzen, 1991). This is in line with Ghani et al. (2013), Stoeva and Alriksson (2017), Liao, Zhao, Zhang, and Chen (2018), Vassanadumrongdee and Suthirat (2018), and Chen and Lee (2020), who highlighted the significant role of attitudes. The term “subjective norm” refers to social pressure influencing individual decisions about whether or not to engage in certain behaviors. PBC is defined by Ajzen and Madden (1986) as “the degree of difficulty an individual perceives in executing a specific behavior”, such as gathering information and associated skills, affordability, and skills. Strydom (2018) examined TPB for waste recycling intentions in South Africa and found that citizens lack adequate understanding, optimistic attitudes, normative influences, and perceived control to promote recycling. Heidari et al. (2018) reported that motivation, followed by moral responsibility, perceived behavior control, subjective norm, situational factor, and attitudes, significantly affect waste segregation intentions.

**Fig. 1:**
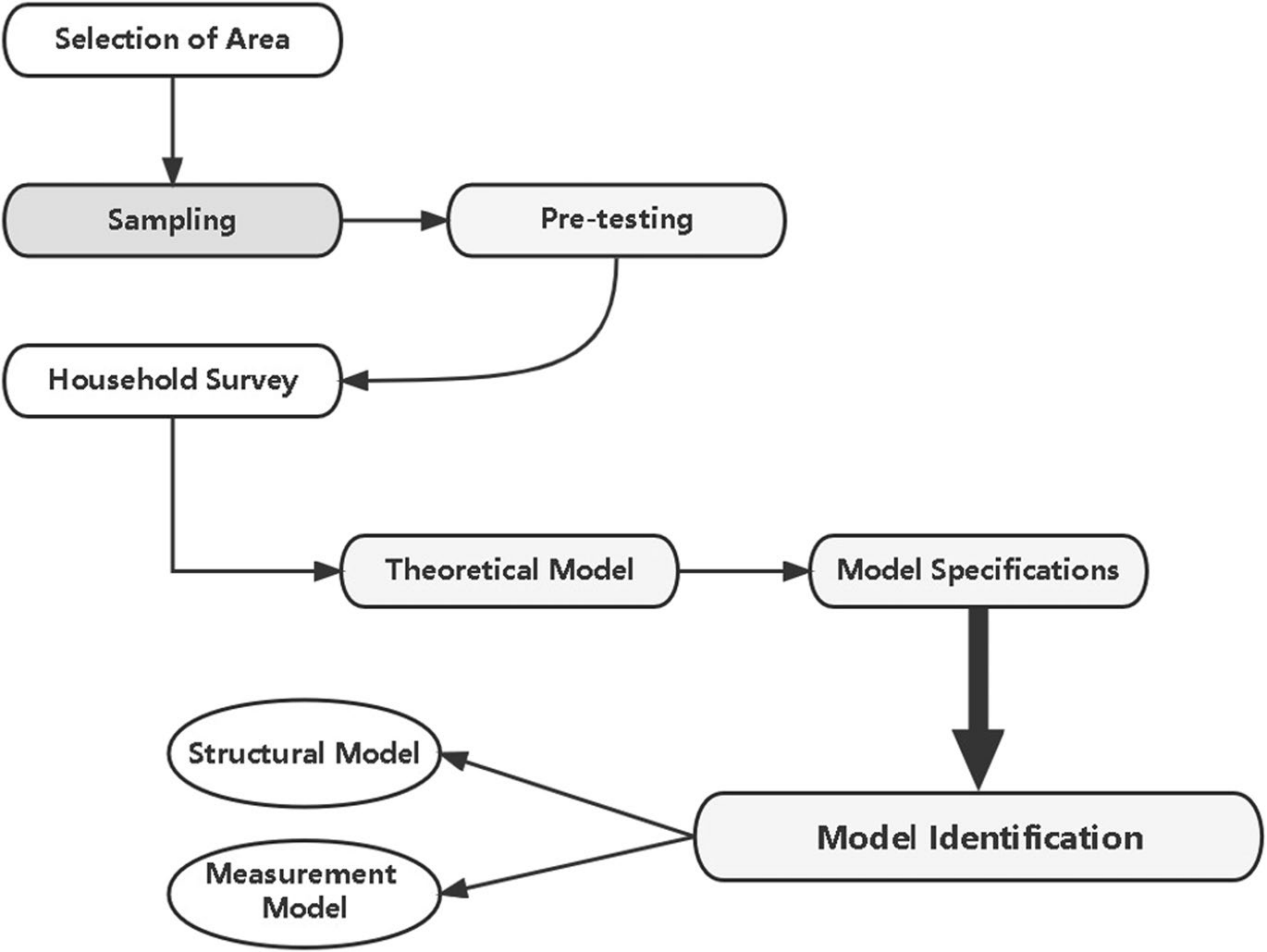
Flowchart of the empirical strategy

In Figure 1 we report the flowchart of the designed methodology.

**Fig. 2:**
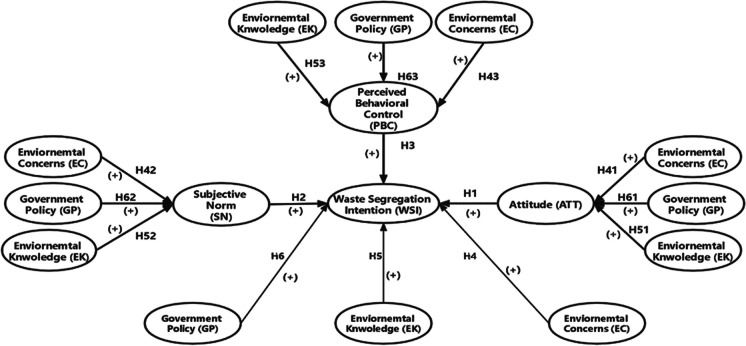
An extended TPB

To drive behavioral change, a mere understanding of TPB constructs is not always sufficient. Instead, understanding the primary determinants that can affect the households’ attitudes, subjective norms, and perceived behavioral control is required (Ajzen, 2011; Bijttebier et al., 2018). However, other latent variables, such as environmental concerns, environmental knowledge, and policies, can be included to measure the validity of TPB (Xu et al., 2017; Xu et al., 2018; Zhang et al., 2015; Zhang et al., 2019). Municipal solid waste management is a public environmental issue influenced by environmental knowledge, which represents an essential element in public environmental issues and social psychology. Additionally, environmental concerns may influence the household’s decision regarding waste segregation and perceived behavioral control. Figure 2 shows the theoretical framework of households’ waste segregation intentions.

### Relevance of transmission channels on TPB constructs

A few studies explored whether external factors, such as government stimulus, environmental concern, and environmental knowledge, would impact waste segregation intention through TPB (Chen & Lee, 2020; Xu et al., 2017; Zhang et al., 2015).

This study investigates the interrelationships among intrinsic factors (attitudes, subjective norms, and perceived behavior control), extrinsic factors (environmental knowledge, environmental concerns, and government policy instruments), and waste segregation intentions. The present research assumes that since the extrinsic factors are recognized as crucial predictors of pro-environmental behavior, it is relevant to understand how residents perceive waste segregation efforts and government stimulus to improve households’ waste segregation intention through these channels. Thus, we added a few constructs of government policy instruments and pro-environmental behavior in the TPB model and explored the role of the TPB model in shaping household segregation behavior, social norms, and ease/difficulty in the presence of external factors.

### Construction of hypotheses

#### Psychological factors

Recycling behavior has essentially been linked to three attributes, including situational, institutional, and psychological variables. Situational factors may include enabling and disabling influences such as environmental knowledge-based (Barr, 2007; Schahn & Holzer, 1990). Personal attitudes, social norms, moral beliefs, and individual perceptions are examples of psychological influences Barr, 2007. Fishbein and Ajzen argued that behavioral intention is driven by immediate determinants like attitudes and subjective norms, since people with high moral and personal norms are more inclined to participate in recycling activities. Ghani et al. (2013), Pakpour et al. (2014), and Xu et al. (2017) confirmed that individual attitudes and waste segregation intention have a positive association. Likewise, Knussen and Yule (2008) and Chen et al. (2019) proved that subjective norms significantly affect households’ recycling behavior. In contrast, Hage et al. (2008) failed to find an association between social influence and recycling behavior. Ajzen (1985) introduced a third predictor of behavioral intention: perceived behavioral control. Wan et al. (2014) identified a positive association between perceived benefits and waste segregation intention. Liao, Zhao, Zhang, and Chen (2018) also confirmed a positive association between Perceived Policy Effectiveness (PPE) and segregation intention. Based on the above literature, the hypothesis is stated as follows:

- H1: ATT has a positive effect on waste segregation intentions
- H2: SN has a positive effect on waste segregation intentions
- H3: PBC has a positive effect on waste segregation intentions.

#### Situational factors

Environmental concern is a key factor in determining the robust attitude to protect the environment, and it triggers environment-friendly attitudes, subjective norms, and perceived behavior control. This, in turn, motivates waste segregation intention. Relating the waste problem to global environmental deterioration increases the willingness to reduce waste production and reuse. This backward extension to the research framework helps to broaden our understanding of social dynamics and their relevance to waste segregation. Thus, these hypotheses are stated as follows.

- H4: EC has a positive effect on waste segregation intentions
- H4_1_: EC does have an indirect positive relationship between a household’s ATT and WSI
- H4_2_: EC does have an indirect positive relationship between a household’s SN and WSI
- H4_3_: EC does have an indirect positive relationship between a household’s PBC and WSI.

Situational factors are characterized by an individual situation at a specific time, such as access to waste management facilities or knowledge of segregation. Guagnano et al. (1995) argued that such factors determine recycling behavior by interacting with specific psychological factors. Knowledge is a prerequisite for engaging in particular behaviors. The relationship between recycling knowledge and recycling behavior has been addressed in several studies (Nixon & Saphores, 2009; Seacat & Northrup, 2010). Li et al. (2019) showed that environmental knowledge and environmental behavior are positively connected.

- H5: EK has a positive effect on waste segregation intentions
- H5_1_: EK does have an indirect positive relationship between a household’s ATT and WSI
- H5_2_: EK does have an indirect positive relationship between a household’s SN and WSI
- H5_3_: EK does have an indirect positive relationship between a household’s PBC and WSI.

#### Institutional factors

The government’s policy instruments are regarded as an institutional factor. They can assist in implementing formal segregation facilities to reap significant economic benefits. Households only participate in segregation activities if they receive monetary incentives or if the government penalizes them (Tonglet et al., 2004; Xu et al., 2017). Chen & Lee, 2020 found that policy regulations are the most important determinant of attitudes in establishing waste segregation intentions. Similarly, Yau (2010) conducted a household survey to understand the household’s willingness to return batteries, highlighting that refundable deposits are a promising factor in deterring households’ behavior in Hong Kong.

Public policy instruments are only effective when policies are premised on a comprehensive understanding of individual behavior and intentions. Therefore, this research focuses on the impact of peer-approved waste segregation policies on household attitudes, social norms, and perceived behavioral intentions. Monetary incentives are mainly daily necessities provided by policymakers who behave well in waste segregation programs; social praise is the public reward for the best behaviors. Thus, we can predict that households are more likely to participate in specific behavior when more incentives are provided. Motivating policies can positively impact the relationship between behavioral intention, attitudes, and perceived behavioral control. Therefore, the following hypotheses are formulated to understand the policy’s direct and indirect effects.

- H6: GPI has a positive influence on waste segregation intentions
- H6_1_: GPI does have an indirect positive relationship between a household’s ATT and WSI
- H6_2_: GPI does have an indirect positive relationship between a household’s SN and WSI
- H6_3_: GPI does have an indirect positive relationship between a household’s PBC and WSI.

## Materials and methods

### Measurement development

The research framework given in Figure 2 contains some latent variables. To measure these latent variables, multiple measurement items are used. To ensure the reliability and validity of each latent variable, the items are all adapted from prior research and appropriately modified to fit the current context of analysis. The measurement items for three behavioral variables, namely attitudes, subjective norms, and perceived behavioral control, are designed following Park and Ha (2014). Three items are used to evaluate each variable. Respondents are asked to analyze these items and express their opinions using a five-point Likert (1932)’s scale ranging from 1 (strongly disagree) to 5 (strongly agree). The items of three additional variables (environmental concern, environmental knowledge, and government policy instruments) are developed following the studies of Wang et al. (2017), Li et al. (2018), and Wang et al. (2020). For item measurements, qualitative scales are defined as follows: 1 = strongly disagree; 2 = disagree; 3 = neutral; 4 = agree; 5 = strongly agree. Three items are constructed on the Likert's scale to evaluate each variable. A variety of rating scales are devised to measure attitudes directly.

The Likert’s scale is the most extensively used method in this kind of research, which assumes that the strength/intensity of an attitude is linear, that is, on a continuum from “strongly agree” to “strongly disagree”, and other variations such as frequency, quality, importance, and likelihood, assuming that attitudes can be measured. Thus, a classified-point Likert-type scale questionnaire is used to collect data on participants’ bio-data, waste disposal, environmental concern, environmental knowledge, waste disposal behavior, and government policy instruments. Category-wise details of items are given in Table 1, which provides the basis of the variables’ construction. Wang et al. (2020) suggested that the waste composition should be known to enable respondents to evaluate their waste sorting intentions, and the waste to be sorted should be specific. The respondents are asked to judge their intentions to sort rubbish in their daily routine. Thus, residents’ waste sorting intention was measured by four items.

**Table 1:** Constructs statements and descriptive states

Items	Items loading	Mean	CR	AVE	Cronbach’s *α*
ATT_1	0.86	3.05	0.70	0.87	0.88
ATT_2	0.81				
ATT_3	0.84				
SNS_1	0.83	2.95	0.77	0.91	0.91
SNS_2	0.96				
SNS_3	0.85				
PBC_1	0.93	3.49	0.70	0.90	0.90
PBC_2	0.88				
PBC_3	0.82				
PBC_4	0.70				
EK_1	0.84	3.06	0.89	0.60	0.89
EK_2	0.66				
EK_3	0.94				
EK_4	0.69				
EK_5	0.77				
EK_6	0.69				
EC_1	0.84	3.49	0.73	0.93	0.94
EC_2	0.82				
EC_3	0.86				
EC_4	0.94				
EC_5	0.83				
GP_1	0.77	3.52	0.70	0.87	0.85
GP_2	0.92				
GP_3	0.81				
WSI_1	0.59	3.99	0.59	0.87	0.85
WSI_2	0.66				
WSI_3	0.96				
WSI_4	0.80				

### Case study area and selection criteria

The case study approach is helpful in an in-depth analysis of the topic by covering the socio-economic, demographic, political, and cultural aspects. This approach is especially suitable when data on the characteristics of the phenomena is missing in the national census data. The cities of Islamabad and Rawalpindi are selected as case studies. Two authorities manage solid waste in these cities, namely Capital Development Authority (CDA) and Rawalpindi Waste Management Company (RWMC).

Islamabad is a well-planned modern metropolis with a grid structure and improved sanitation system. On the contrary, Rawalpindi is an old city with an unplanned web structure. Because of its intricate construction design, the city is more congested and has a poor sanitation system. The total urban area of the twin cities is 3,723 km^2^. Islamabad is predominantly urban, and 95% of its population lives in urban areas, compared to 63% of the total population of Rawalpindi (Rehman & Jamil, 2021).

The diversity of the population is mainly due to being the federal capital and our sample comprises representation from all four provinces living in the twin cities. Therefore, it makes a more insightful case study, which may help to formulate the policy design for the entire country. Furthermore, as the population growth rate in the twin cities is higher than the national average, this ever-increasing population in the study area puts pressure on respective municipalities and sanitation systems, leading to inadequate planning and design. Islamabad has a well-operated WMS compared to all other cities of Pakistan. An estimated 60% of waste is collected and transferred to the dumping site. However, sanitary landfill sites are not constructed; thereby, open dumping is a widely used method all over the country. The daily average waste collection rate in Islamabad is 900-1,000 tons; 1.89 kilograms per house is the waste produced in Islamabad, and 0.8 in Rawalpindi (ADB, 2022). In Rawalpindi, throwing waste into sewage watercourses (Nalah) is a regrettable standard practice. People used to dispose of their household waste at Nalah in many places due to the absence of other arrangements that obstruct water flow or cause groundwater contamination. In addition, irregular waste disposal sites are seen within most of the residential localities in Rawalpindi and several areas of Islamabad. So that it seems appropriate to select this region as a case study.

### Survey design and data collection

Data on household segregation intention was gathered to achieve the research’s objectives. Primary data comprises structured interviews with 849 respondents randomly selected from 35 residential areas of the twin cities to test the hypotheses. Out of 35 residential areas, 15 sites were chosen from Islamabad and 20 from Rawalpindi. Further, these towns/sectors are divided based on planned and unplanned regions. The sample size is calculated following Cochran (1977). This study uses a semi-structured questionnaire for interviewing the selected respondents who are either household heads or main female members of the household because they manage the waste, and their intention is most relevant in waste segregation. The questionnaire is constructed under the theoretical framework and considers factors measuring the determinants isolated in the previous waste disposal and environment-related studies (Choon et al., 2016; Zhang et al., 2015).

Meanwhile, these measurement items were slightly revisited and refined to fit the current research context. Several factors have been considered, such as the socio-economic and demographic characteristics of selected households in the survey. After conducting a pre-testing in the field, a questionnaire was finalized that helped to better contextualize the idea and revise it as needed. Details about the items for each variable are provided in the Appendix (Table 8).

## Data analysis and results

SPSS 22.0 software was used for statistical analysis. Table 1 shows that the means of government policy instruments (3.52), environmental concerns (3.49), and environmental knowledge (3.06) are relatively high. It is worth noting that the mean values of external variables are higher than those of internal variables. This research validated the discrepancy between internal and external waste segregation intentions and indicated that external factors are essential in shaping households’ self-motivated behavior.

Furthermore, Table 1 also highlights that the factor loading value of each measurement item is greater than 0.70 and that each item is significantly loaded to the related latent variable. The reliability and validity were tested via Composite Reliability (CR), Convergent Validity (CV), and Discriminant Validity (DV). Further, the study adopted Anderson and Gerbing (1988)’s two-step approach to test the model. The first step is to achieve a satisfactory Measurement Model (MM) using Confirmatory Factor Analysis (CFA). The second phase involves developing and testing the Structural Model (SM). The results of Cronbach’s *α* values for seven latent variables are as follows: ATT (0.88), SN (0.91), PBC (0.90), EC (0.94), EK (0.89), GPI (0.85), and WSI (0.85), revealing good internal consistency. The results indicate that all factor loading values are statistically significant at the 1% level of significance.

### Socio-economic characteristics of the sample

1,000 households were initially selected for the study, and 151 refused to participate. A total of 849 households were finally included in the study. The majority of respondents (64.90%) in the sample are female because they generally manage the waste in the house, and around 93% of the respondents are between 15 and 40 years old. Around 24% of the respondents are illiterate, 7.42% have completed primary level, 24.50% have completed secondary level, 22.15% have completed high school education, and 21.67% have acquired a university degree. The majority of respondents belong to the medium-income group (45.47%), followed by the low (30.27%), and high-income groups (24.26%). Descriptive statistics are given in Table 2.

**Table 2:** Demographic information of respondents

Demographic characteristic	Frequency	Percentage
Gender		
Female	551	64.90%
Male	298	35.10%
Age		
15-20	245	28.86%
21-30	315	37.10%
31-40	228	26.85%
41-50	50	5.89%
> 51	11	1.30%
Education		
Illiterate	206	24.26%
Primary School	63	7.42%
Secondary School	208	24.50%
High School	188	22.15%
University	184	21.67%
Income levels		
Low income	Middle income	High income
257 (30.27%)	386 (45.47%)	206 (24.26%)

### Measurement model

Construct validity was tested by measuring CV and DV. The CV of standardized factor loadings on each latent variable was examined by checking their magnitudes, direction, and statistical relevance. In addition, CV was analyzed using the Average Variance Extracted (AVE) and the Composite Reliability (CR). MM is valid when a minimum level of AVE is higher than the 50% level, and the minimum value of CR is higher than 0.7. DV was measured by comparing the AVE assessments for the latent variable respectively with the squared inter-construct correlations related to that latent construct. AVE for each construct should be greater than the squared inter-construct (Hair et al., 2010). Results of AVE and Cronbach’s *α* values for latent variables are given in Table 1. The results indicate that all factor loading values are statistically significant at a 1% level. Factors loading suggest an acceptable CV. In addition, the results of the correlation between constructs are given in Table 9 (see the Appendix).

CFA was applied to check the properties of the measurement scale (see Table 8). It shows goodness-of-fit and specific indices for the empirical data (Bagozzi, 1994). The *χ*^2^ standardized by degrees of freedom is = 5.46. The Normed Fit Index (NFI) and Comparative Fit Index (CFI) are 0.90 and 0.91, respectively, for an indirect effect of behavioral attributes in model 2, whereas *χ*^2^ is 3.77. NFI and CFI are 0.90 and 0.95 respectively, for indirect effects in the second transmission model of TPB. The results of CFA suggest that both models are adequate for the empirical analysis (see Table 3).

**Table 3:** Reliability and validity test

Fit Index	TPB Model (M_1)		Transmission channels of TPB Model (M_2)	
	Rec. Value	SM (Results)	Rec. Value	SM (Results)
*χ*^2^ test statistics	>3.00	5.46	>3.00	3.77
CFI	>0.90	0.91	>0.90	0.95
NFI	>0.90	0.90	>0.90	0.93
RMSEA	<0.08	0.073	<0.08	0.057

Rec. Value (Recommended Value) (*χ*^2^ should be < 5 (Bentler, 1990); RMSEA (Root Mean Square Error of Approximation) should be < 0.10 (Henry & Stone, 1994). CFI Comparative Fit Index, NFI Normed Fit Index

### SEM results

Once we obtain a valid MM, the next step is to test the empirical proposed framework, the Maximum Likelihood-Structural Equation Modeling (ML-SEM), using AMOS software. Previous research has primarily focused on understanding whether various strategies can promote waste segregation, with little attention paid to the underlying mechanisms that underpin policy intervention effects, resulting in a shaky link between intervention-based (awareness, incentives) and psychological factors (Varotto & Spagnolli, 2017). In order to fill the research gap, policy instruments, environmental knowledge, and social pressure are considered as the direct paths to increase waste segregation. The first model explains the direct relationship between these social and economic variables (see Table 8). The SEM results demonstrate that attitudes positively influence household waste segregation intentions and, thus, support H_1_. Our findings are similar to several previous studies (Echegaray & Hansstein, 2016; Liao, Zhao, & Zhang, 2018; Williams & Taylor, 2004; Xu et al., 2017). However, few studies found a weak relationship between attitudes and recycling intentions (Martin et al., 2006; Strydom, 2018), which supports the argument that a positive relationship between attitudes and recycling intentions does not always lead to actual behavior.

The primary purpose of model (2) is to investigate the indirect effect of environmental knowledge, environmental concern, and government policy instrument on households’ attitudes, subjective norms, and perceived behavior control to waste segregation intention. Notwithstanding, prior research has not fully addressed the role of external stimuli as a moderator in the relationship between traditional social attributes and waste segregation intentions. As a result, this paper is projected to broaden the researchers’ understanding of the specific interrelationships between these variables. Therefore, we further examine whether the indirect effect of policy variables on ATT, SN, and PBC exists for waste segregation intentions. The estimated standardized coefficients between the latent variables in model (2) are shown in Table 4.

**Table 4:** SEM results of an extended TPB

Hypothesis	Standardized estimate	Std. Errs.	Result
ATT → WSI	0.112***	0.027	Not rejected
SN → WSI	0.136***	0.026	Not rejected
PBC → WSI	0.060**	0.026	Not rejected
EC → WSI	0.034**	0.017	Not rejected
EK → WSI	0.137***	0.020	Not rejected
GPI → WSI	0.377***	0.041	Not rejected

*p < 0.01, **p < 0.05, ***p < 0.001

### Discussion

The empirical findings of this research reveal that subjective norms have the most decisive impact on households’ waste intentions (coefficient = 0.136, p < 0.01), followed by attitudes (coefficient = 0.112, p < 0.01) and perceived behavioral control (coefficient= 0.060, p < 0.05), implying that the role of social norms on waste segregation intention is inevitable (Strydom, 2018) and, hence, support H_2_. The results are consistent with Wan et al. (2014), Ari and Yilmaz (2016), and Shen et al. (2019), who found that subjective norms are an important predictor of recycling. On the other hand, Ayob et al. (2017), indicated that subjective norms are insignificant determinants of waste segregation intention. Overall, this finding suggests that households’ intention to waste segregation is highly dependent on their personal motivation and confidence; it is influenced by the opinions of the people who are important to them, such as family and friends. Furthermore, perceived behavior control is also a significant predictor of intention to waste segregation and, thus, supports H_3_. Xu et al. (2017) and Echegaray and Hansstein (2016) provided a significant relationship between PBC and waste segregation intentions. On the contrary, Strydom (2018) and Khan et al. (2019) found that PBC is an insignificant predictor of waste segregation.

Our findings extend the traditional social model by exposing the diverse impacts of social persuasion in incentive-based settings. Therefore, the present study results reveal that environmental knowledge, environmental concern, and government policy directly influence households’ waste segregation intentions. Environmental concern (coefficient = 0.034, p < 0.05) is positively related to waste segregation intention, which verifies the null hypothesis H_4_. Jekria and Daud (2016) also reported a positive relationship between environmental concern and recycling behavior. On the other hand, Ng et al. (2021) showed an insignificant relationship between environmental concern and intentions for food waste segregation. Likewise, environmental knowledge (coefficient = 0.137, p < 0.01) positively affects waste segregation intention, which confirms H_5_. Results imply that knowledge of what can be recycled or reused and how to do that represents a real determinant of the dependent variable, so information campaigns should be directed at the household’s level. However, the previous literature has questioned the explanatory link between environmental knowledge and pro-environmental behavior Bamberg & Möser, 2007. More specifically, the link between environmental knowledge and environmental behavior has been shown to be significant but relatively weak (Frick et al., 2004). In addition, Paco and Lavrador (2017) explained that the increase in knowledge, which merely emphasizes the pro-environmental domain, has not always been successfully translated into actual pro-environmental behavior.

Furthermore, government policy (coefficient = 0.377, p < 0.01) is the most powerful indicator of households’ waste segregation intentions among the external stimulation factors, which verifies H_6_. Results align with existing literature (Bernstad, 2014; Chen & Lee, 2020; Wan et al., 2014). It is also possible to note that households’ waste segregation intentions could become mainstream with the strengthening effect of government support. Government policy instruments enable the implementation of waste segregation behavior much easier. It also helps in the transformation from household intentions to actual behavior. In this respect, Agovino et al. (2018) showed that citizens’ waste management behavior would be improved if the government pursued joint action. Likewise, Dos Muchangos et al. (2015) confirmed that policy implementation in Mozambique experienced several obstacles and significantly impacted waste management efficiency. Several studies in Malaysia have found that loopholes in policy enforcement in solid waste management cause national serious environmental problems (Abas & Wee, 2014; Moh & Abd Manaf, 2014). A similar issue emerges in Cameroon and many other African cities, where regulatory execution has been inadequate, resulting in rising waste pollution (Manga et al., 2008). On the other hand, Lapinski et al. (2017) found that external incentives distort the positive impact of social norms on environmental behavior.

Moving to model (2), the standardized coefficients of ATT, SN, and PBC results improve to (0.169, 0.185, and 0.233, respectively) but remain statistically significant at the same significance level as in model (1).

Furthermore, the results imply that personal attitudes influence the progression from intention to waste segregation behavior, social pressure, moral morbidity, and the ease or difficulty of the action. This result does not support the motivation crowded-out theory, which explains that incentive-based strategy distorts the effect of social norms (Rode et al., 2015; Varotto & Spagnolli, 2017). These findings posit that external attributes positively impact waste segregation intentions via social and personal traits. Both models show that an economic stimulus and social influence may effectively increase waste segregation. It must be noted that when institutional and situational elements are effectively channeled through the social construct, perceived behavioral control emerges as the most influential factor in driving waste segregation intentions. The importance of perceived behavioral control confirms that households’ ability to recycle is influenced by other social factors, e.g., government policy, environmental knowledge, and environmental concerns. Government policies have the most deceptive impact on PBC among external factors.

Environmental concerns are indirectly related to waste segregation intentions via attitudes (coefficient = 0.063, p < 0.01) and perceived behavior control (coefficient = 0.090, p < 0.01), which supports H4_1_ and H4_3_. Table 5 shows that attitudes have a strong indirect relationship between environmental concern and waste segregation intention at the 1% significant level. Jekria and Daud (2016) found a significant positive indirect association between environmental concerns and recycling behavior via attitudes. Ng et al. (2021) highlighted that households with environmental concerns demonstrate “positive environmental attitudes, subjective norms, and perceived behavioral control”, resulting in high food waste segregation intentions. However, this is not true for the subjective norms, where the coefficient (-0.004) is statistically insignificant and inconsistent with H4_2_. It means that environmental concerns do not have an anticipatively positive relationship with segregation intention via subjective norms.

**Table 5:** Transmission channels’ effects on TPB structures

Hypothesis	Standardized estimate	Std. Errs.	Result
ATT → WSI	0.169***	0.030	Not rejected
SN → WSI	0.185***	0.029	Not rejected
PBC → WSI	0.223***	0.024	Not rejected
EK → ATT	0.106***	0.028	Not rejected
EK → SN	0.180***	0.029	Not rejected
EK → PBC	0.016	0.028	Rejected
GP → ATT	0.328***	0.041	Not rejected
GP → SN	0.488***	0.045	Not rejected
GP → PBC	1.010***	0.050	Not rejected
EC → ATT	0.063***	0.024	Not rejected
EC → SN	-0.004	0.024	Rejected
EC → PBC	0.090***	0.024	Not rejected

NS Not Significant. *p < 0.10, **p < 0.05, ***p < 0.01

The results of the indirect effect also pinpoint that environmental knowledge has a significant indirect effect on waste segregation intention through attitudes (coefficient = 0.106, p < 0.01) and subjective norms (coefficient = 0.180, p < 0.01). Hence, environmental knowledge indirectly affects waste segregation intention, which supports H5_1_ and H5_2_. These findings imply that attitudes and subjective norms are mainly influenced by the awareness of consequences, which represents an individual tendency to relate their behavior to the welfare of others, and the ascription of responsibility, which refers to taking responsibility for behavioral consequences. Results are partially in line with those by Park and Ha (2014) and Wang et al. (2020), who highlighted a significant indirect effect between environmental knowledge and waste sorting attention through attitudes and subjective norms. On the other hand, Arkorful et al. (2021) indicated an insignificant relationship between environmental knowledge attitude and mask segregation intention.

The significance of government policy instruments as a moderator in the relationship between conventional social attributes and waste segregation intentions has been thoroughly examined in prior studies. Unlike social behavior alone, the integrated model revealed that the effects of personal attitudes, social norms, and perceived severity on segregation intention are influenced by government policy. Government policy instruments are indirectly associated with waste segregation intentions via subjective norms (coefficient = 0.488, p < 0.01), as well as by attitudes (coefficient = 0.328 p < 0.01), and perceived behavioral control (coefficient = 1.010, p < 0.01). Thus, government policies have the most substantial intrinsic and extrinsic impact on household waste segregation intentions. Therefore, H6_1_, H6_2,_ and H6_3_ support that government policy instruments positively affect social-behavioral constructs and waste segregation intention. It implies that the government’s monetary incentives, fines, and coercion would help to persuade the households to improve their personal and social behavior regarding waste segregation. These findings indicate that a behavioral adjustment is required before enforcing waste segregation policies. In this respect, this study’s results align with those of Saphores and Nixon (2014), Liao, Zhao, Zhang, and Chen (2018), and Wang et al. (2020).

In general, the results explain that behavior is likely to be affected by personal motivation and other factors such as environmental knowledge, environmental concerns, and government policy instruments. In addition, perceived behavioral control appears to have a greater influence on waste segregation intention. Moreover, results show that the indirect effect of social-incentive constructs outweighs the direct impact. Particularly, perceived behavior control has a weaker direct effect on household waste segregation intentions than an indirect effect, implying that situational and institutional factors may be used to decide if a particular cognitive choice is enough. Our empirical findings also highlight that an increased government supervision and the development of environmental protection infrastructure are essential factors in improving households’ segregation intentions.

### Robustness checks: Unpooled data results

As further robustness checks, the results of unpooled data for model (1) provide an intriguing interpretation (see Table 6). In fact, the estimates show that the standardized coefficient of ATT is a stronger predictor for Rawalpindi than Islamabad. SN has a positive effect on waste segregation intentions in Islamabad at a significant 5% level, although this is not true in the case of Rawalpindi. Disaggregated results show additional interesting insights; PBC has a high impact in determining waste segregation intentions for Rawalpindi, which implies that here the households are confident they could segregate waste if proper waste separation facilities and knowledge are provided. The PBC coefficient (0.348) indicates that people’s behavioral intentions are highly reliant on their ability to regulate themselves. Since residents do not have waste separation facilities, municipalities must provide segregation facilities for public assistance to make it easier. Likewise, government policy (coefficient = 0.149, p < 0.01) is a significant positive predictor of waste segregation intentions in Rawalpindi.

**Table 6:** SEM results of an extended TPB for unpooled data

Hypothesis	Standardized estimate (Rawalpindi)	Results (Rawalpindi)	Standardized estimate (Islamabad)	Results (Islamabad)
ATT → WSI	0.147*** (0.035)	Not rejected	0.098*** (0.039)	Not rejected
SN → WSI	0.059* (0.033)	Rejected	0.109*** (0.036)	Not rejected
PBC → WSI	0.348*** (0.043)	Not rejected	-0.006 (0.033)	Rejected
EC → WSI	-0.001 (0.019)	Rejected	-0.047* (0.026)	Rejected
EK → WSI	0.211*** (0.031)	Not rejected	0.073*** (0.025)	Not rejected
GPI → WSI	0.149*** (0.054)	Not rejected	-0.006 (0.033)	Not rejected

Standard Errors in parentheses. *p < 0.10, **p < 0.05, ***p < 0.01

On the other hand, PBC has an insignificant impact on waste segregation intentions in Islamabad (coefficient = -0.006). These results are in line with Chen and Tung (2010). While interviewing them, we found that respondents were willing to pay for waste segregation rather than being involved in waste segregation activity due to time constraints. Another explanation for this behavior is the household’s income. Most of the respondents in the Islamabad area were in the high- or medium-income group. Likewise, government policy (coefficient = -0.006) is not statistically significant to predict waste segregation intention in Islamabad. A possible reason for this result is that WMS in Islamabad is comparably better, and the majority of residents have a door-to-door collection system. Because the norm has been established, policy tools may be ineffective in Islamabad. This conclusion provides policymakers with food for thought in terms of determining which segments of the population should be targeted and what type of policy might be helpful in achieving the desired goals.

The indirect effects of the social incentive model on waste segregation intentions for the unpooled data set have been also estimated (see Table 7). The findings show that households who are concerned about the environment have positive attitudes toward pro-environmental behavior. GPI is the strongest indirect predictor of waste segregation intention among external and internal drivers in both cities. Likewise, an indirect effect of EK on waste segregation intentions also has a significant impact via TPB constructs.

**Table 7:** Transmission channels effects on TPB structures for unpooled data

Hypothesis	Standardized estimate (Rawalpindi)	Results (Rawalpindi)	Standardized estimate (Islamabad)	Results (Islamabad)
ATT → WSI	0.060 (0.040)	Rejected	0.216*** (0.040)	Not rejected
SN → WSI	0.111*** (0.037)	Not rejected	0.120*** (0.036)	Not rejected
PBC → WSI	-0.075** (0.037)	Not rejected	0.409*** (0.071)	Not rejected
EK → PBC	-0.131*** (0.040)	Not rejected	0.103*** (0.035)	Not Rejected
EK → ATT	-0.075** (0.036)	Not rejected	0.335*** (0.046)	Not rejected
EK → SN	-0.027 (0.038)	Rejected	0.417*** (0.050)	Not rejected
GP → PBC	0.387*** (0.061)	Not rejected	0.977*** (0.064)	Not rejected
GP → ATT	0.273*** (0.056)	Not rejected	0.207*** (0.056)	Not rejected
GP → SN	0.387*** (0.061)	Not rejected	0.423*** (0.062)	Not rejected
EC → PBC	0.062 (0.041)	Not rejected	0.142*** (0.025)	Not rejected
EC → ATT	0.049 (0.037)	Rejected	0.070** (0.029)	Not rejected
EC → SN	-0.124*** (0.039)	Not rejected	0.040 (0.031)	Rejected

Standard Errors in parentheses. *p < 0.10, **p < 0.05, ***p < 0.01

Contrary to Rawalpindi, TPB constructs do not predict the behavioral intentions of the metropolitan of Islamabad; however, SN has a positive and significant effect on waste segregation intention. PBC shows a negative and meaningful relationship with waste segregation intention and contradicts the earlier work of Visschers et al. (2020). The results also show that behaviors regarding waste segregation are driven more by subjective norms than by their capacity and convenience to do so (Zhang et al., 2019). In addition, the indirect effect of environmental knowledge has a negative and significant impact on waste segregation intention through attitudes and PBC. The negative coefficient of environmental knowledge indicates that, even though environmental knowledge is an important factor in influencing waste segregation intention, it is insufficient to persuade a group of individuals to engage in pro-environmental action.

## Conclusion

This study provides empirical evidence of the relationship among policy instruments, social norms, and personal attitudes within the waste segregation domain. Applying an SEM to a representative sample comprising 849 households in two cities in Pakistan (Islamabad and Rawalpindi), we found that attitudes, subjective norms, perceived behavioral control, environmental concerns, government policy, and environmental knowledge significantly affect household waste segregation intentions in Pakistan. Furthermore, the findings revealed that economic incentives based on households’ performance would encourage waste segregation intention, which might eventually translate into real practice. The results further indicated that environmental concerns, government policy, and environmental knowledge impact waste segregation intention directly and indirectly via attitudes, subjective norms, and perceived behavioral control. The effective transformation of a household’s perceived responsibility to personal and social norms into actual participation in the provision of public goods is significantly reliant on public policy (Mele et al., 2022). Moreover, government policy instruments about waste segregation have the highest direct impact on households’ waste segregation intentions. This analysis of different institutional, situational, and psychological factors can also help to develop targeted policy interventions to achieve sustainable waste management (Magazzino et al., 2021b; Magazzino & Mele, 2021). Furthermore, environmental knowledge emerged as a decisive driver in waste segregation behavior, and results confirmed its significant positive effect on attitudes and subjective norms. Hence the study proves the effectiveness of social attributes in understanding the households’ waste segregation behavioral intentions.

## Policy implications, limitations, and future research

These results have useful policy insights for policymakers in establishing a trajectory toward a circular economy. We suggest that the government’s monetary incentives, coercion, and penalties should be devised in the early phases of a waste separation design to motivate waste segregation in the twin cities. Moreover, residents of Rawalpindi are motivated to begin a waste segregation behavior via incentives, while residents in Islamabad are motivated by social influences.

The important implication for the household’s involvement in the waste segregation program requires more knowledge, which is again necessary for assisting CDA^1^ and RWMC^2^ officials (who are directly involved with households in implementing any policy at the field level). There is a need to improve social awareness among the members of society: this effort should start at the school level, and moral obligations campaigns should carry out aiming to improve environmental knowledge and individuals’ waste separation abilities.

Finally, this study examined the intention towards waste segregation application rather than the actual behavior of a household. We acknowledge as a limitation of this study that other factors (that were not considered in the survey) might influence actual behavior between the phase when an intention is formed and when it is fully implemented. Thus, in this respect, future studies may focus on identifying whether the households’ waste segregation intentions might be translated into actual practice or not. The results of this study can be used as a reference to those analyses. In fact, this study helps to explain how the extended TPB model affects households’ intentions and predicts each individual behavior. Households are more likely to adopt market-related factors. The proposed framework did not consider those market-related factors. Therefore, to find households’ intentions toward waste segregation, adding psychological, economic, and financial factors (e.g., selling recyclables, income, health incentives, and environmental benefits) is recommended. In addition, the analysis of latent variables might be performed with innovative Artificial Intelligence tools (Magazzino et al., 2020).

## Data Availability

Data published in this study are available on request from the corresponding author. The data are not publicly available due to the policy of the research project.

## Appendix

**Table 8:** Questionnaire

Item	Responses					Source
**Attitudes**						
Waste segregation helps conserve resources and the environment	1	2	3	4	5	Zhang et al., 2017; Xu et al., 2017
In my opinion, waste segregation is a good idea.	1	2	3	4	5	Liao, Zhao, Zhang, & Chen, 2018
In my opinion, the segregation of waste is essential for hygiene.	1	2	3	4	5	Liao, Zhao, Zhang, & Chen, 2018
**Subjective norms**						
My family and friends are doing MSWS.	1	2	3	4	5	Xu et al., 2017
My family thinks segregation helps minimize the environmental effects.	1	2	3	4	5	Yu et al., 2018
I value the opinion of my family on the segregation of Waste	1	2	3	4	5	López-Mosquera et al., 2014
**Perceived behavior control**						
MSWS is effortless.	1	2	3	4	5	Zhang et al., 2015
I have enough time for waste segregation.	1	2	3	4	5	Zhang et al., 2015
I have complete awareness; what items should be segregated.	1	2	3	4	5	Xu et al., 2017
I have all the necessary resources for segregation that allow me segregation.	1	2	3	4	5	López-Mosquera et al., 2014
**Environmental knowledge**						
Household waste segregation can bring economic benefits.	1	2	3	4	5	Chen & Lee, 2020
Household Segregation of waste can help to enhance landfill life.	1	2	3	4	5	Wan et al., 2014
Household waste segregation can help to decrease the morbidity rate.	1	2	3	4	5	
Household waste segregation can minimize environmental damages.	1	2	3	4	5	Chen & Lee, 2020
I am aware of the need to reduce waste for environmental protection in the long run.	1	2	3	4	5	Chen & Lee, 2020
I am aware of the possible link between disease symptoms and improper waste disposal.	1	2	3	4	5	
**Environmental concerns**						
Environmental changes in the world are my biggest concern.	1	2	3	4	5	
Dumpsite near my house is my primary concern.	1	2	3	4	5	Chen & Tung, 2014
Improper waste blocking severely affects my aesthetic sense.	1	2	3	4	5	
We should care about water contamination issues due to long-term open dumping.	1	2	3	4	5	
I often think about how the environmental quality in Pakistan can be improved.	1	2	3	4	5	Tonglet et al., 2004
**Government policy instruments**						
Do you support the government incentivizing (penalty/ rewards) residents to encourage waste segregation activities?	1	2	3	4	5	Xu et al., 2017
Are the separation facilities (3 bins waste collection) provided by the government sufficient to facilitate segregation?	1	2	3	4	5	Xu et al., 2017
Do you support the government in developing laws and regulations regarding waste separation and environmental awareness?	1	2	3	4	5	Chen & Lee, 2020
**Waste segregation intentions**						
I intend to segregate waste to avoid its adverse effects on the environment.	1	2	3	4	5	
I suffered from waste-related diseases, so I intend to do waste segregation.	1	2	3	4	5	
I intend to recover resources responsibly (e.g., WTE, composting).	1	2	3	4	5	
I will try to segregate waste over the next month to minimize the morbidity rate.	1	2	3	4	5	Xu et al., 2017

Notes: 1= Strongly Disagree; 2= Disagree; 3=Netural; 4= Agree; 5=strongly Agree.

**Table 9:** Correlation matrix

	ATT	SN	PBC	EK	EC	GPI	WSI
ATT	1						
SN	0.341** (0.001)	1					
PBC	0.258** (0.001)	0.429** (0.000)	1				
EK	0.158** (0.00)	0.233** (0.76)	0.118** (0.001)	1			
EC	0.112** (0.001)	0.017 (0.631)	0.126** (0.00))	0.051 (0.134)	1		
GPI	0.270** (0.000)	0.379** (0.000)	0.651** (0.000)	0.137** (0.000)	0.460 (0.181)	1	
WSI	0.258** (0.000)	0.325** (0.000)	0.375** (0.000)	0.299** (0.000)	0.137** (0.000)	0.457** (0.000)	1

** significant at a 1% level.

## Footnotes

1 Capital Development Authority is a public benefit corporation responsible for providing municipal services in Islamabad Capital Territory.

2 Rawalpindi waste Management Company is responsible for providing municipal services in Rawalpindi.
